# Chronic wasting disease alters the movement behavior and habitat use of mule deer during clinical stages of infection

**DOI:** 10.1002/ece3.11418

**Published:** 2024-05-21

**Authors:** Gabriel M. Barrile, Paul C. Cross, Cheyenne Stewart, Jennifer Malmberg, Rhiannon P. Jakopak, Justin Binfet, Kevin L. Monteith, Brandon Werner, Jessica Jennings‐Gaines, Jerod A. Merkle

**Affiliations:** ^1^ Department of Zoology and Physiology University of Wyoming Laramie Wyoming USA; ^2^ U.S. Geological Survey, Northern Rocky Mountain Science Center Bozeman Montana USA; ^3^ Wyoming Game and Fish Department Jackson Wyoming USA; ^4^ Department of Veterinary Sciences University of Wyoming Laramie Wyoming USA; ^5^ USDA‐APHIS, Wildlife Services, National Wildlife Research Center Fort Collins Colorado USA; ^6^ Haub School of Environment and Natural Resources University of Wyoming Laramie Wyoming USA; ^7^ Haub School of Environment and Natural Resources, Department of Zoology and Physiology, Wyoming Cooperative Fish and Wildlife Research Unit University of Wyoming Laramie Wyoming USA

**Keywords:** Bayesian inference, behavioral change, cause‐specific mortality, *Cervidae*, chronic wasting disease, host–pathogen dynamics, space use, transmissible spongiform encephalopathy

## Abstract

Integrating host movement and pathogen data is a central issue in wildlife disease ecology that will allow for a better understanding of disease transmission. We examined how adult female mule deer (*Odocoileus hemionus*) responded behaviorally to infection with chronic wasting disease (CWD). We compared movement and habitat use of CWD‐infected deer (*n* = 18) to those that succumbed to starvation (and were CWD‐negative by ELISA and IHC; *n* = 8) and others in which CWD was not detected (*n* = 111, including animals that survived the duration of the study) using GPS collar data from two distinct populations collared in central Wyoming, USA during 2018–2022. CWD and predation were the leading causes of mortality during our study (32/91 deaths attributed to CWD and 27/91 deaths attributed to predation). Deer infected with CWD moved slower and used lower elevation areas closer to rivers in the months preceding death compared with uninfected deer that did not succumb to starvation. Although CWD‐infected deer and those that died of starvation moved at similar speeds during the final months of life, CWD‐infected deer used areas closer to streams with less herbaceous biomass than starved deer. These behavioral differences may allow for the development of predictive models of disease status from movement data, which will be useful to supplement field and laboratory diagnostics or when mortalities cannot be quickly retrieved to assess cause‐specific mortality. Furthermore, identifying individuals who are sick before predation events could help to assess the extent to which disease mortality is compensatory with predation. Finally, infected animals began to slow down around 4 months prior to death from CWD. Our approach for detecting the timing of infection‐induced shifts in movement behavior may be useful in application to other disease systems to better understand the response of wildlife to infectious disease.

## Graphical abstract

Our manuscript characterizes how infection with chronic wasting disease (CWD) alters the movement and habitat use of free‐ranging mule deer. We demonstrate that movement data (i.e., locations from GPS collars) are predictive of disease status as early as 4–6 months prior to death from CWD. We further demonstrate that behavioral changes (e.g., decreased movement speed, increased use of riparian habitat) in CWD‐infected deer can be distinguished from similar behavioral shifts in other pathologic states such as malnutrition and starvation.

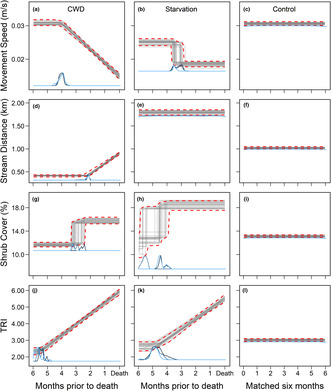

## 1 INTRODUCTION

Emerging infectious diseases threaten global biodiversity and present major challenges for wildlife management (Jones et al., 2008). Consequently, there is growing recognition that evaluating how wild animals behave in response to infection is needed to better understand the epidemiology of emerging pathogens, predict changes to host distribution, assess transmission risk, and ultimately develop control measures (Brannelly et al., 2021; Edmunds et al., 2018). For instance, animals can be constrained to, manipulated toward, or actively select certain habitats when infected, which alters host distribution and can increase transmission risk (and/or predation risk) in such environments (Dobson, 1988; Holmes & Zohar, 1990). Amphibians infected with chytrid fungus (*Batrachochytrium dendrobatidis*) shift to warm, dry, open habitats (Barrile et al., 2021; Daversa et al., 2018), arthropods infected with *Cordyceps* fungi climb to the extremities of plants before death (Roy et al., 2006), and rodents infected with *Toxoplasma gondii* spend more time in exposed areas after losing their fear of predators (Berdoy et al., 2000; Gatkowska et al., 2012). Infection with disease also can alter the movement behavior of hosts. Infection with sarcoptic mange (*Sarcoptes scabiei*), for instance, can decrease daily movements in wolves (*Canis lupus*; Cross et al., 2016) and contribute to differences in home range size in coyotes (*Canis latrans*; Murray et al., 2015). Taken together, characterizing how infection influences host movement and habitat use will be critical for understanding the ecology of emerging pathogens and for developing effective management strategies for species challenged by disease.

Yet, linking host behavior and disease dynamics remains a central issue in wildlife disease ecology (Manlove et al., 2022). Rigorous integration necessitates concomitant measurements of individual space use and infection status, both of which can be difficult to sample in wild populations (Ryser‐Degiorgis, 2013). For instance, disease status is often known only intermittently because it typically requires animals to be captured. Detecting infection‐induced shifts in host behavior is especially challenging for chronic diseases, moreover, as infected individuals may not exhibit conspicuous changes in behavior during prolonged latent periods of infection (Williams, 2005). Thus, characterizing how animals respond behaviorally to infection with chronic diseases can require following many animals and repeatedly testing them for extended periods of time (Cross et al., 2009; Wobeser, 2007). For these reasons, the influence of chronic diseases on host movement and habitat use is poorly understood.

We capitalized on cause‐specific mortality data from mule deer (*Odocoileus hemionus*) fitted with GPS collars to investigate how movement behavior and habitat use may be influenced by infection with a chronic disease. Chronic wasting disease (CWD) is a transmissible spongiform encephalopathy that affects members of the *Cervidae* family (Miller et al., 2000; Williams & Young, 1980) with the potential for population‐level consequences (DeVivo et al., 2017; Edmunds et al., 2016). The impacts of CWD may be more severe in certain species and environments, such as less productive semi‐arid systems (Foley et al., 2016). CWD can be transmitted through direct animal contact (Williams et al., 2002) and via environmental contamination (Almberg et al., 2011; Mathiason et al., 2009), as infectious prions—the causative agent of CWD—can persist for years to decades in the environment (Miller et al., 2000). Notably, CWD can alter normal host behavior (Williams, 2005). Captive cervids exhibit behavioral alterations during clinical phases of infection, which can occur over a few weeks to a few months before death (e.g., 2 weeks–8 months before death; Williams & Young, 1980). Such changes in behavior include lethargy, reduced alertness, head tremors, ataxia, listlessness, and erratic behavior (Fox et al., 2006; Williams & Young, 1980). Late‐stage CWD also can cause polydipsia (i.e., extreme thirst), such that infected cervids may shift habitat use in search of drinking water (Miller et al., 1998; Williams & Young, 1993).

Documentation of behavioral change in CWD‐infected hosts is almost exclusively limited to observations of captive animals. To our knowledge, only one published study directly examined how CWD influences the movement and habitat use of wild cervids. Edmunds et al. (2018) demonstrated that CWD‐positive animals were less active throughout the day than CWD‐negative individuals in a population of free‐ranging white‐tailed deer (*Odocoileus virginianus*). Further, the average clinical period of CWD infection was 16.25 weeks, though only four deer died of clinical CWD while equipped with GPS collars (Edmunds et al., 2018). Thus, current inference from wild populations relies on a small sample size, and it remains unclear how other deer species may respond behaviorally to CWD infection.

We evaluated the extent to which CWD infection alters host behavior in two populations of mule deer in central Wyoming. GPS collars on a subset of the population (*n* = 179) provided relocations every 1–2 h, and cause of death was determined for most collared animals that experienced mortality. We compared movement and habitat use of deer that died from CWD to those that succumbed to starvation (and were CWD‐negative) and others in which CWD was not detected (i.e., deer that died from causes other than CWD or starvation and deer that survived the duration of the study). Further, we conducted a time‐series analysis on infected individuals to identify both the timing of behavioral change and the average duration of overt clinical disease. We anticipated that shifts in movement behavior and habitat use would occur approximately 16 weeks before death from CWD (Edmunds et al., 2018). Further, given extreme thirst during the late stages of CWD, we expected infected deer to move closer to water sources in the months preceding death (Miller et al., 1998). We also expected infected animals to become lethargic, behave more erratically (e.g., tortuous movements), and use areas with less of their preferred forage as neurodegeneration progressed (Fox et al., 2006; Williams & Young, 1980). We discuss our results in the context of disease management, with specific emphasis on predator cleansing, environmental clean‐up, and identifying potential hotspots for transmission. Finally, we discuss the potential for future work to capitalize on the differences in behavior between infected and uninfected conspecifics to create predictive models for improved disease surveillance (Dougherty et al., 2018).

## 2 METHODS

### 2.1 Study area

**FIGURE 1:**
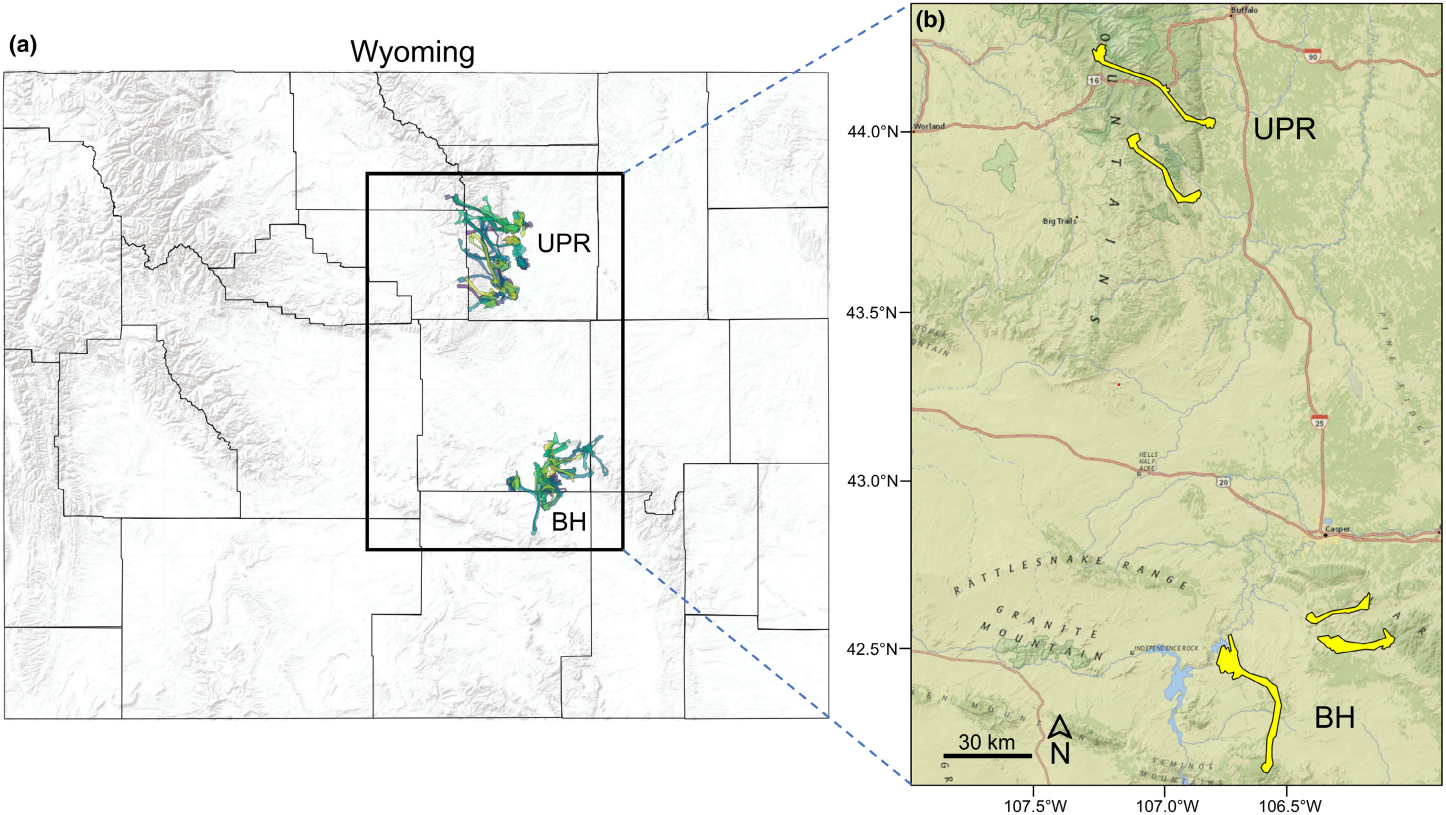
(a) We analyzed the movement behavior and habitat use of adult female mule deer (*n* = 179) collared during 2018–2022 in a CWD‐endemic region of central Wyoming, USA. Colored polygons in (a) denote home ranges for each deer. We focused our analysis on two distinct populations: the Bates Hole (BH) herd south of Casper, Wyoming and the Upper Powder River (UPR) herd south of Buffalo, Wyoming. Yellow polygons in (b) display home ranges for a few deer with representative migrations in each population.

We studied mule deer within a CWD‐endemic region of central Wyoming, USA (Figure 1). Based on surveillance of hunter‐harvested deer, CWD prevalence in this region was 28.4% during 2020–2022 (Wyoming Game and Fish Department, 2023). We focused our analysis on two populations: the Bates Hole/Hat Six (hereafter, Bates Hole) herd south of Casper, Wyoming (~6000 km^2^; 42°39′ N, 106°27′ W) and the Upper Powder River herd south of Buffalo, Wyoming (~7000 km^2^; 43°57′ N, 106°51′ W) (Figure 1a). Both populations occupied terrain typical of foothill habitat found in the Rocky Mountain West (Decker, 2007); the landscape was a matrix of mixed‐conifer and aspen (*Populus tremuloides*) forests, mixed mountain shrub communities, sagebrush (*Artemisia* spp.) and grassland prairie, and riparian areas dominated by cottonwood (*Populus* spp.), box elder (*Acer negundo*), and willow (*Salix* spp.). The regional climate is characterized by long, cold winters and relatively short, warm summers. Mean daily temperatures ranged from −6°C in January to 20°C in July, with a mean annual precipitation of 384 mm (climate data summarized between 1900 and 2022 from NOAA Online Weather Data, National Oceanic and Atmospheric Administration).

### 2.2 GPS collar data

We captured mule deer on winter range via helicopter net‐gunning in accordance with https://wgfd.wyo.gov/WGFD/media/content/Habitat/WY‐Deer‐Capture‐Protocol_Final_v2022.pdf. Depending on the procedures performed, deer were administered light sedation, injectable and/or topical analgesics, or a combination thereof under the guidance of a licensed veterinarian. Vitals including temperature, pulse, and respiration were monitored and recorded during handling and all capture and handling methods were approved by an Institutional Animal Care and Use Committee (Protocol # 20210303KM00462 for Bates Hole and # 18–02 for Upper Powder River), and aligned with animal handling guidelines (Sikes, 2016).

Captured animals were fit with GPS store‐on‐board collars (Bates Hole: Vectronic Aerospace, Berlin, Germany; Upper Powder River: Telonics (TGW‐4560‐4), Mesa, Arizona, USA). GPS units obtained a fix every hour for deer in Bates Hole and every 2 h for deer in Upper Powder River.

We collated GPS data for adult (>1.5 year old) females collared during 2021–2022 in Bates Hole (*n* = 64) and 2018–2021 in Upper Powder River (*n* = 115). Adult males were not collared during these efforts, and we excluded fawns from analysis because juvenile deer rarely test positive for CWD (Miller & Conner, 2005). We excluded deer with collar malfunctions and removed all additional erroneous fixes from the dataset before analysis. Specifically, we removed points far outside the study area and those representing unreasonably high movement speeds (>14 km/hr).

Collar data revealed a mixture of resident and migratory individuals in both populations. Migratory deer in Bates Hole moved between winter ranges in the lower‐lying areas of Bates Hole (elevation = ~1800 m) to summer ranges in either the Shirley Mountains to the south (~2700 m) or the northern Laramie Mountains to the east‐northeast (~2400 m) (Figure 1b). Migratory deer in Upper Powder River moved between winter ranges just west of Interstate 25 (between the towns of Kaycee to the south and Buffalo to the north; elevation = ~1500 m) to summer ranges in the southern Bighorn Mountains to the west‐northwest (~2700 m) (Figure 1b). We determined the proportion of CWD‐infected deer that were migratory in comparison with the migratory proportion of all other deer (i.e., deer that did not die from CWD). We defined migration as movement between distinct winter and summer ranges, irrespective of distance traveled. Thus, we classified migratory individuals simply by visually inspecting their GPS data.

### 2.3 Cause‐specific mortality

GPS units were equipped with a mortality signal that activated after 10 h of inactivity. We responded to mortalities in effort to determine cause of death based on field observations, gross and histopathologic assessment of available tissue, and laboratory testing. Response time was variable and postmortem condition of carcasses ranged from excellent to poor due to scavenging and/or autolysis. We collected detailed information about the site of the mortality and the condition of the carcass and performed necropsies, typically in the field using a standardized protocol, with tissues collected and submitted to a board‐certified pathologist at the Wyoming State Veterinary Laboratory for gross and histologic evaluation, body condition assessment, and determination of CWD status. We also performed PCR for viral hemorrhagic diseases (i.e., deer adenovirus, epizootic hemorrhagic disease virus, and bluetongue virus), bacterial culture, hepatic trace element panels, and tooth aging, all contingent on available tissues. CWD diagnostics included enzyme‐linked immunosorbent assay (Hibler et al., 2003) on one medial retropharyngeal lymph node and immunohistochemistry (Miller & Williams, 2002) on obex, tonsil, and the contralateral retropharyngeal lymph node as available. Body condition assessment included gross and histologic evaluation of bone marrow for serous atrophy of fat. When carcasses or other tissues were available, we also assessed fat deposits elsewhere (e.g., retroperitoneal, retrobulbar, and epicardial). We recorded cause of death for each mortality regardless of CWD status at the time of necropsy.

Ultimately, we split cause of death into five categories: CWD, starvation (i.e., emaciation in a CWD‐negative animal without evidence of other disease processes), predation (subdivided into predator type; Cristescu et al., 2022), euthanasia, and other (including trauma, vehicle collision, poaching, and harvest). Death was attributed to CWD when a deer tested positive and no other cause of death or concurrent pathologic process was identified. Given that CWD has been shown to increase host vulnerability to predators (Miller et al., 2008), we additionally included deer that tested positive for CWD and were killed by a predator (*n* = 6, individuals 62, 71, and 81 in the Upper Powder River herd and D018, D020, and D033 in Bates Hole) in the CWD category for our analyses. When possible, we confirmed histologic lesions indicative of clinical CWD (i.e., spongiform change in the brain). However, histologic confirmation was not always possible due to autolysis or scavenging. Deer assigned to the predation category were those in which evidence of predation was identified in the absence of a predisposing pathologic process, as determined by a board‐certified pathologist. For simplicity, hereafter we refer to deer assigned to the CWD category as “infected” and all other deer as “uninfected.”

Although 32 adult females were assigned CWD as the cause of death, 14 of those deer had been collared less than 6 months before death. Given that we aimed to characterize and identify the timing of infection‐induced behavioral change, we only included animals with greater than 6 months of GPS data in our analyses of movement and habitat use. Moreover, only 12 CWD‐positive deer were collared for more than 8 months before death, thus we restricted analyses to the 6 months preceding death to retain an adequate sample size of CWD‐positive deer (*n* = 18; 8 in Bates Hole and 10 in Upper Powder River). Finally, to ensure that postmortem relocations were not included in the analysis, we removed GPS fixes within 24 h of death for all individuals that experienced mortality.

### 2.4 Movement metrics

We calculated movement speed, turning angle, and displacement between consecutive relocations for each deer. Displacement denoted the total distance traveled (in m), speed signified the distance traveled divided by time elapsed (in m/s), and relative turning angle represented the change in direction between successive steps (in radians). To reduce spatiotemporal autocorrelation among telemetry locations, we summarized movement parameters as daily metrics (i.e., one value per day for each deer). We calculated both mean and maximum speed per day, displacement measured the maximum distance between any two relocations for an individual during a given day, and turning angle constituted a daily average.

### 2.5 Habitat covariates

In conjunction with movement behavior, we expected CWD infection to alter habitat use during the late stages of disease. We therefore intersected GPS relocations with vegetation cover, topographic, hydrographic, and anthropogenic variables relevant to mule deer ecology (Dwinnell et al., 2019; Lendrum et al., 2012). All environmental variables were derived from remotely sensed geospatial data layers at 30‐m resolution.

We downloaded vegetation cover variables from the Rangeland Analysis Platform (RAP v3.0; https://rangelands.app/), which provides annual numeric estimates of herbaceous biomass (grasses + forbs) and percent cover values for various vegetation cover types (e.g., grasses, shrubs, trees). To derive topographic features, we first imported digital elevation models from the National Elevation Dataset (U.S. Geological Survey, 2022) using the *get*_*ned* function within the “FedData” package in Program R version 4.3.2 (R Core Team, 2023). Elevation layers were then used to calculate various topographic metrics using functions within the “spatialEco” package in R (Evans & Ram, 2021). Specifically, we calculated slope and terrain ruggedness index, which quantified topographic heterogeneity (Riley et al., 1999). We also created variables for distance to roads and linear and area hydrography using the *roads*, *linear_water*, and *area_water* functions within the “tigris” package (Walker, 2021). Whereas the linear hydrography shapefile included streams/rivers, braided streams, canals, ditches, and aqueducts, the area hydrography included larger waterbodies such as ponds, lakes, and reservoirs.

### 2.6 Behavior of infected versus uninfected deer

We anticipated deer that succumbed to starvation may behave similarly to CWD‐infected deer (e.g., lethargy, decreased movement speed), particularly toward the end of life. Thus, we divided our dataset into three groups during all analyses described below: (1) deer that died from CWD (i.e., “infected” deer), (2) deer that died from starvation (i.e., emaciation in a CWD‐negative animal without evidence of other disease processes), and (3) all remaining deer, including animals that died from other causes and deer that remained alive at the end of the study. Deer that died from starvation served as a positive control, whereas “all remaining deer” served as a negative control.

To determine how CWD might alter the behavior of mule deer, we compared daily metrics of movement and habitat use between the three groups described above using three separate conditional logistic regression procedures. First, we compared behavioral metrics between CWD‐infected deer and negative control deer (response variable = infected [coded as 1] and negative control [coded as 0]). Second, we compared metrics between deer that died of starvation (positive control) and negative control deer (response variable = starved [coded as 1] and negative control [coded as 0]). Third, we compared metrics between CWD‐infected deer and positive control deer (response variable = infected [coded as 1] and starved [coded as 0]).

We employed conditional logistic regression to restrict behavioral comparisons between groups to those within the same herd and during the same time of year (i.e., a stratum included only data from the same week of the year and population). Such conditional comparisons accounted for natural differences in behavior throughout the annual cycle (e.g., animals move more quickly during migration; habitat availability differs between summer and winter ranges). Conditional comparisons also corrected for any potential bias in natural behavior if CWD and/or starved animals died more often during a specific time of year. Importantly, our approach assumed habitat availability was similar for individuals within the same herd during the same time of year.

**FIGURE 2:**
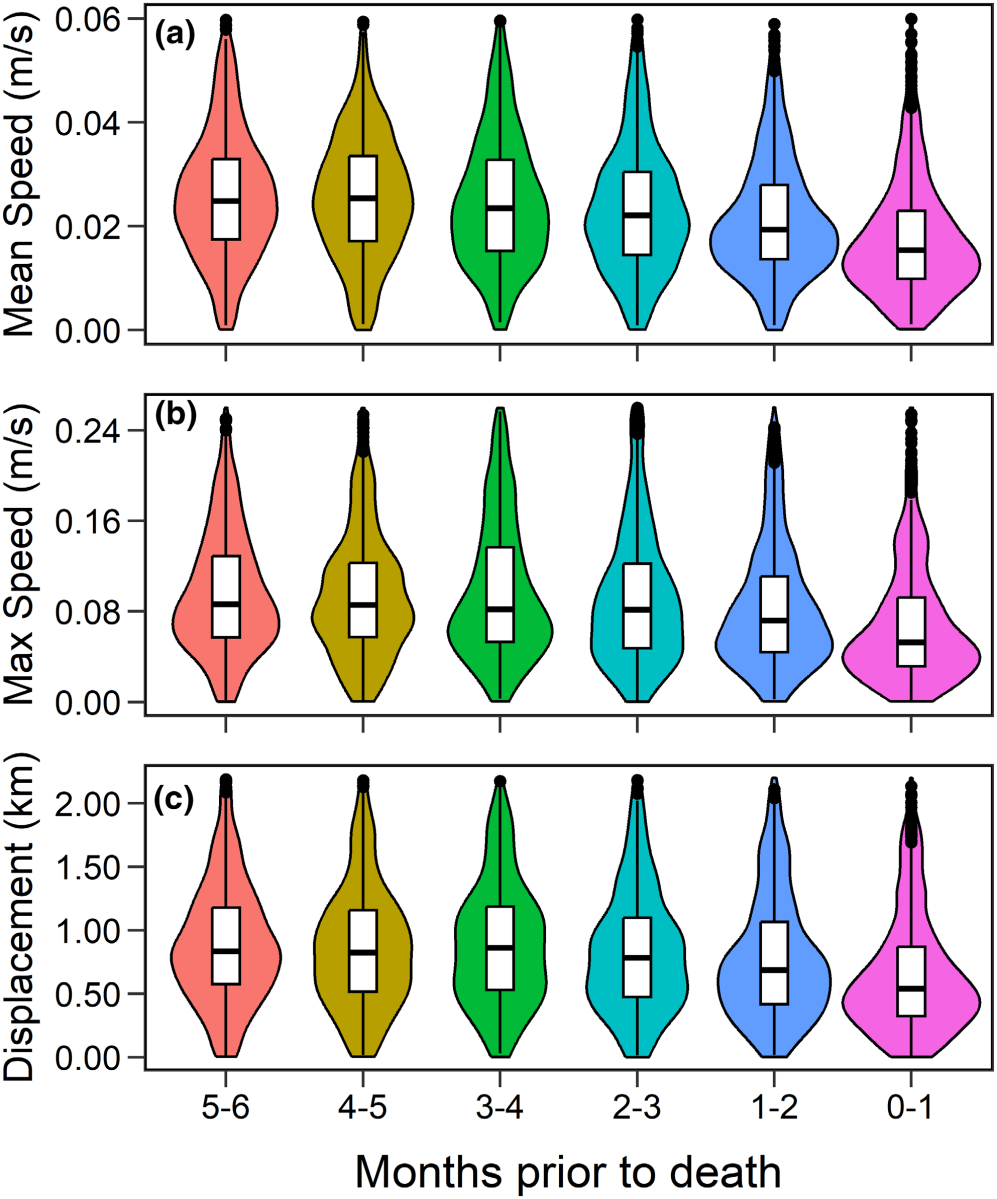
CWD‐infected mule deer (*n* = 18) moved at relatively slower speeds (a, b) and shorter distances (c) as death from CWD approached within a sample of adult females collared during 2018–2022 in central Wyoming, USA. Violin plots display the distribution of the raw data, including the density of each movement metric: (a) mean daily movement speed; (b) maximum daily movement speed; and (c) displacement, which measured the maximum distance between any two relocations for an individual during a given day. The widths of violins are proportional to the approximate frequency of data points in each region. Boxplots display quartiles of the raw data, with black dots representing potential outliers.

We removed correlated variables (Pearson's correlation coefficients > |0.60|) before fitting final models (Appendix S1: Table S1). To determine which correlated variables to retain for analysis, we compared univariate models (among each set of correlated variables) with Akaike information criterion and retained the variable that produced the model with the lowest Akaike information criterion score (Burnham et al., 2011). Ultimately, we removed the variables of displacement, maximum speed, slope, percent cover of forbs and grasses, and proximity to larger water bodies (e.g., lakes, reservoirs) (Appendix S1: Table S2). For instance, mean movement speed was highly correlated with maximum movement speed and displacement. We used mean movement speed in analyses because it produced a better model fit (Appendix S1: Table S2), though raw data suggest that these three variables followed similar patterns during the final months of life in CWD‐infected animals (Figure 2).

We then parameterized full models with all remaining movement and habitat variables (nine total covariates; all nine covariates appear in Figure 4, except for relative turning angle). In the first model procedure (infected versus negative control), given that we were uncertain when the behavior of infected animals may deviate from other animals, we fit a separate model for each month before death from CWD, using data from the final 6 months of life for infected deer. In other words, we partitioned data from infected deer into 1‐month time bins over the last 6 months of life (i.e., 6 bins) and temporally matched data from deer in the negative control to each bin (i.e., subset data in the negative control to match the same year, month, and day of data points from infected animals). For example, if infected animals had zero GPS fixes on June 5, 2019, then those data points were removed from the negative control prior to analysis. We then parameterized six models, one for each month before death.

For the second model procedure (starved vs. negative control, again with six total models), we repeated the methods described in the previous paragraph, replacing infected deer with those that died from starvation. Notably, given that no animals died from starvation in Bates Hole during our study, only data from the Upper Powder River were included in analyses with starved deer.

The third conditional logistic procedure (infected vs. starved, six total models) differed slightly from the first two. Rather than temporally matching negative control data to the exact day/month/year of case data, we compared behavioral metrics during the final 6 months of life between infected and starved animals. Nevertheless, behavioral comparisons between infected and starved deer also were restricted to animals within the same herd and during the same time of year. In other words, like model specification for the first two procedures, a stratum in these conditional logistic models included all observations from CWD‐infected and starved individuals within the same herd and during the same week of the year. Again, given that no animals died from starvation in Bates Hole, only data from the Upper Powder River herd were included in this analysis. To account for spatiotemporal autocorrelation and pseudoreplication among an individual's telemetry locations, we calculated robust standard errors and 95% confidence intervals of parameters using generalized estimating equations (Craiu et al., 2008). All observations for a given individual were assigned a unique cluster (Merkle et al., 2014). Model parameters were estimated using maximum likelihood in the “survival” package (*clogit* function) in Program R. We centered (by subtracting variable means) and scaled (by dividing centered variables by their standard deviations) variables to better compare their relative importance with the response (Schielzeth, 2010).

We structured the statistical models described above with individual “states” (infected, starved, negative control) as the response variable (rather than behavioral metrics) to include multiple movement and habitat use covariates within the same model and compare their relative effect sizes. When interpreting model results (see Results and Discussion), we infer and discuss differences in behavior between infected, starved, and negative control animals. However, we acknowledge that models were structured as behavioral metrics predicting individual states, rather than states predicting behavior. Thus, we do not ascribe causality to states being predictive of behavior (e.g., CWD status predicts movement speed) (Kimmel et al., 2021), but maintain that models were structured to simultaneously evaluate potential differences in multiple behavioral metrics among infected, starved, and negative control animals.

### 2.7 Timing of behavioral change in infected deer

Piecewise regression can identify change points that signify shifts in the relationship between response (e.g., movement speed) and predictor variables (e.g., time until death from CWD) and allows for unique model structures for each segment between change points (Toms & Lesperance, 2003; Toms & Villard, 2015). Transitions between segments can be smooth if segments are joined (share a common endpoint), or abrupt, if segments are disjoint (do not share a common endpoint) (Wolfson et al., 2022).

To identify the potential presence and timing of infection‐induced shifts in behavior of CWD‐infected deer, we conducted piecewise regression to partition movement and habitat use patterns into multiple segments using time‐series data during the final 6 months of life. We repeated this analysis for deer that died from starvation, again using data during the final 6 months of life for those individuals. We also repeated the analysis with negative control animals (i.e., deer that died from causes other than CWD or starvation and those that survived the duration of the study). Given that some negative control animals did not experience mortality, we could not focus on the final 6 months of life in this analysis. Instead, for each CWD‐infected and starved deer, we temporally matched data from a negative control deer and conducted piecewise regression on this dataset. For instance, if a CWD‐infected deer's final 6 months spanned September–February, then we subset data during September–February in a random negative control deer. Such temporal matching—rather than sampling a random, consecutive 6‐month period from each negative control deer—corrected for any potential bias in natural behavior if CWD and/or starved animals died more often during a specific time of year (e.g., deer may naturally slow down during winter months, irrespective of disease status). We expected negative control deer to exhibit no behavioral change and thus serve as an appropriate control.

For each movement and habitat variable included in the final conditional logistic models (see the *Behavior of Infected* vs. *Uninfected Deer* section above), we fit three piecewise regression models corresponding to specific biological hypotheses: (1) single intercept with slope = 0, which represented a null model of no behavioral change; (2) two disjointed intercepts, both with slope = 0, which represented an abrupt shift between two behavioral states (e.g., onset of clinical CWD caused sudden change in behavior); and (3) intercept and slope = 0 in the first segment with a joined slope sharing the same intercept in the second segment, which represented consistent behavior (i.e., plateau) followed by a gradual shift in movement or habitat use, progressively increasing or decreasing as death from CWD or starvation approached.

We performed piecewise regression by fitting Bayesian hierarchical models in the “mcp” package (Lindeløv, 2020). Within hierarchical models, we specified random effects to allow change points to covary for each individual deer, thereby allowing the estimation of individual‐specific and population‐level change point parameters. During model checking, we conducted posterior checks using the *pp_check* and *plot_pars* functions (e.g., to visualize chain mixing for change point parameters) in the “mcp” package and visualized posterior fit using q_fit = TRUE when plotting inferred models (Conn et al., 2018). We also checked that Gelman–Rubin diagnostics were below 1.1 as an indicator that MCMC chains converged well (Gelman & Rubin, 1992). Then, to evaluate the relative support for each hypothesis described above, we compared models with leave‐one‐out cross‐validation using the Estimated Log‐Predictive Density (ELPD), as estimated by the “loo” package (Vehtari et al., 2017). ELPD indicates how much credence the model ascribes to out‐of‐sample data and hence is a relative index of predictive performance (Gelman et al., 2014). Absolute ELPD values are difficult to interpret, however, thus we calculated the difference in ELPD between models, and then divided by the standard error of said difference. This ratio (referred to below as ELPD:SE) is analogous to a z‐score, wherein a ratio of 1.96 corresponds to 95% probability that one model has superior predictive accuracy.

## 3 RESULTS

### 3.1 CWD‐infected deer migrated in similar proportions to other deer

We collared 179 adult females (64 deer at Bates Hole and 115 in Upper Powder River) during this study, of which 151 were collared for more than 6 months (58 deer at Bates Hole and 93 in Upper Powder River) and thus included in analysis. Migratory deer typically began spring migration to higher elevation summer ranges in May and returned during autumn migration in October and November. Approximately 60% were migratory in Bates Hole (35/58) compared with 34% in Upper Powder River (32/93). Within each herd, CWD‐infected deer migrated at similar proportions to all other deer: 62% (5/8) of infected versus 60% (30/50) of uninfected deer migrated at Bates Hole and 40% (4/10) of infected versus 34% (28/83) of uninfected deer migrated in Upper Powder River.

### 3.2 CWD was the leading cause of mortality

**FIGURE 3:**
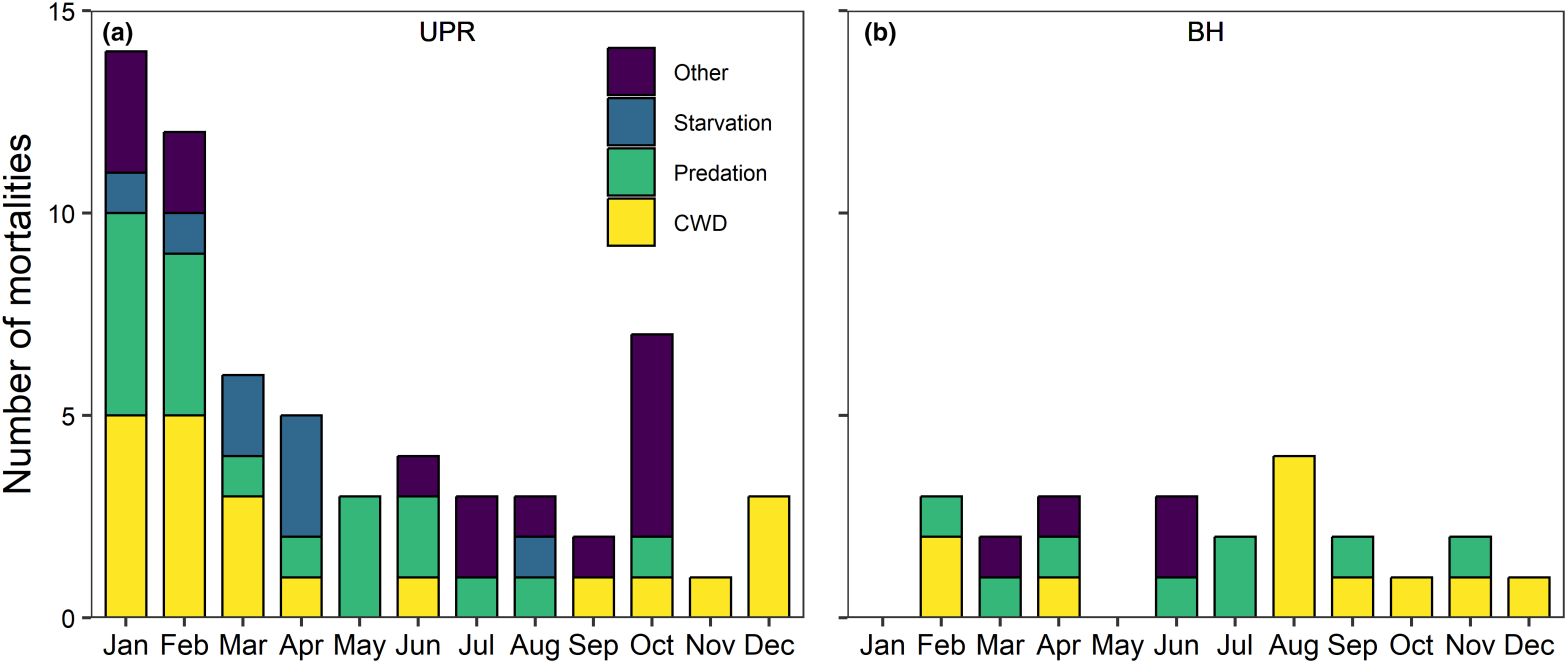
Monthly cause‐specific mortalities of adult female mule deer (*n* = 91) collared during 2018–2021 in (a) Upper Powder River and during 2021–2022 at (b) Bates Hole in a CWD‐endemic region of central Wyoming, USA. Notably, the earliest collaring at Bates Hole was in March 2021, such that mortalities in January and February in (b) only encompass 1 year (2022) whereas other months include both 2021 and 2022. Deer that were euthanized (*n* = 5) in response to injuries during capture do not appear in the figure as euthanasia does not constitute a naturally‐occurring cause of death.

Ninety‐one collared deer (adult females) died during the study period: 32 from CWD, 27 from predation, eight from starvation, five were euthanized, and 19 from other causes, which included trauma, vehicle collision, urinary tract infection, poaching, and harvest (Figure 3). CWD mortalities were concentrated to some degree during December–March, with 19 of 32 (59%) deaths occurring in these 4 months. Notably, only one deer died from CWD during May–July (Figure 3). Predators included coyote (*Canis latrans*), bobcat (*Lynx rufus*), cougar (*Puma concolor*), and black bear (*Ursus americanus*), with cougars accounting for 20 of the 27 kills during our study. All euthanasia occurred in response to injuries during capture (LaSharr et al., 2023), except for one animal caught in a fence. No animals were euthanized due to starvation.

### 3.3 CWD‐infected animals behaved differently than starved and negative control animals

**FIGURE 4:**
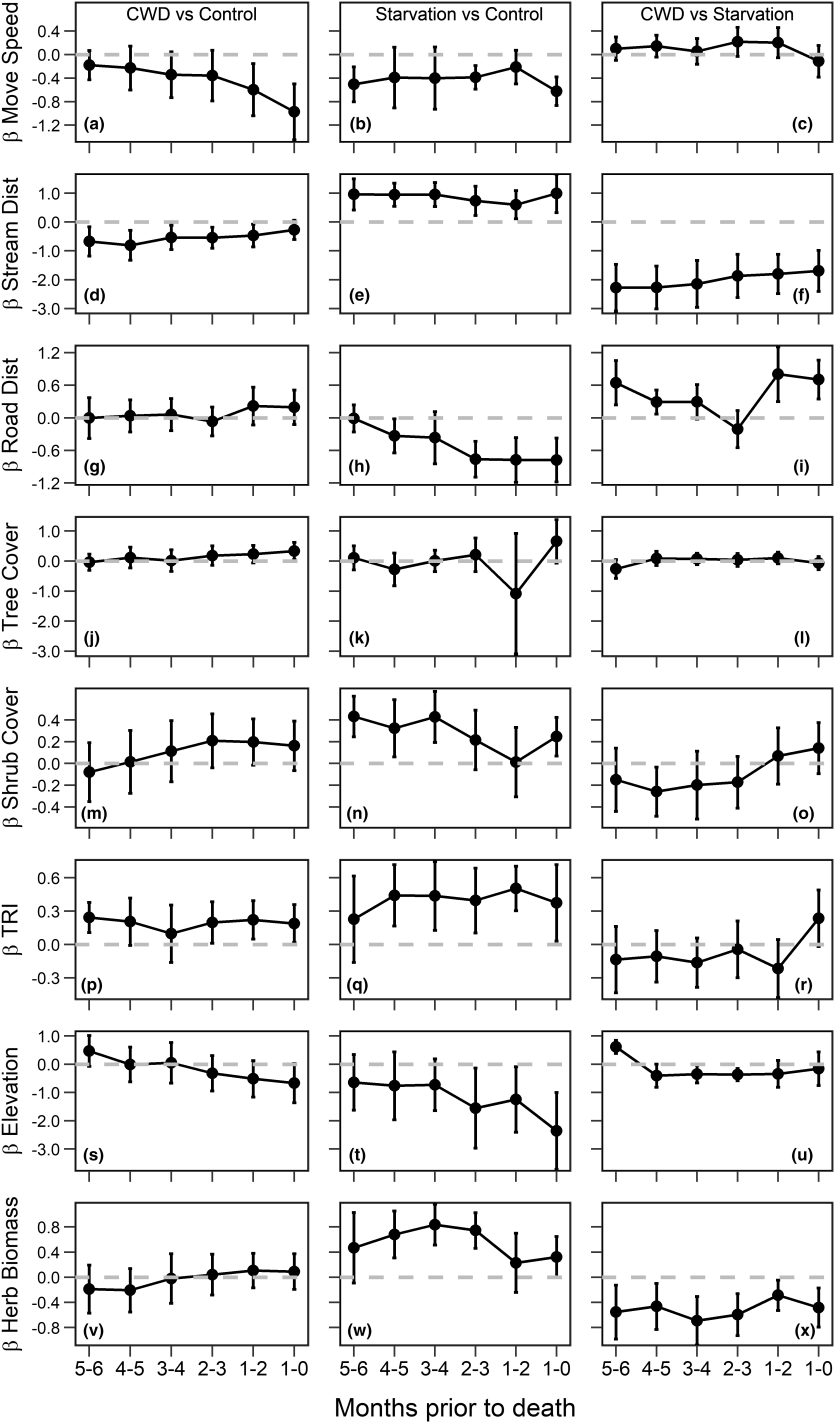
During the final 6 months of life, CWD‐infected mule deer (*n* = 18) often moved and used habitat differently than starved (*n* = 8) and negative control deer (*n* = 127; deer that died from causes other than CWD or starvation and those that remained alive at the end of the study) within a sample of adult females collared during 2018–2022 in a CWD‐endemic region of central Wyoming, USA. Standardized parameter estimates (i.e., scaled slope coefficients; black circles) were derived from conditional logistic regression models that compared behavior between: (1) CWD‐infected and negative control animals (column 1 in the figure: response variable = infected [coded as 1] and negative control [coded as 0]); (2) starved and negative control animals (column 2 in the figure: response variable = starved [coded as 1] and negative control [coded as 0]); and (3) infected and starved animals (column 3 in the figure: response variable = infected [coded as 1] and starved [coded as 0]). The horizontal dashed line in grey at y = 0 denotes no difference in behavior between groups for each variable included in the model. For example, in column 1 of the figure, estimates indicate the direction and extent to which the behavior of CWD‐infected deer differed from negative control animals, whereas column 3 of the figure displays the direction and extent to which the behavior of CWD‐infected deer differed from animals that succumbed to starvation (e.g., negative values in (f) indicate that CWD‐infected deer used areas closer to streams than deer that succumbed to starvation). We derived 95% confidence intervals (error bars) for each parameter using robust standard errors from generalized estimating equations. Notably, given that no animals died from starvation in Bates Hole during our study, only data from the Upper Powder River were included in analyses with starved deer.

CWD‐infected and starved deer did not differ in movement speed (Figure 4c). Both groups moved more slowly than negative control deer, though patterns varied: during the final 6 months of life, starved deer moved at a consistently lower speed than negative control deer (Figure 4b), while CWD‐infected deer slowed to a greater extent as death approached (Figure 4a). CWD‐infected deer used areas closer to streams than both starved (Figure 4f) and negative control deer (Figure 4d), even 6 months prior to death. By contrast, starved deer generally used areas closer to roads than both negative control deer (Figure 4h) and those that died from CWD (Figure 4i). Further, CWD‐infected deer used areas with slightly higher tree cover than negative control deer, though only during the final month of life (Figure 4j). CWD‐infected and starved deer did not differ in their use of tree and shrub cover during the final 6 months of life (Figure 4l,o). CWD‐infected and starved deer also did not differ in their use of elevation (Figure 4u) nor topographic heterogeneity (Figure 4r), though both groups generally used more rugged terrain than negative control deer (Figure 4p,q). CWD‐infected and starved deer also used areas with slightly lower elevations than negative control deer, though only during the final month of life (Figure 4s,t). Finally, starved deer generally used areas with greater herbaceous biomass than both negative control animals (Figure 4w) and those that died of CWD (Figure 4x). Relative turning angle did not differ statistically in any analysis (i.e., infected versus negative control deer, starved versus negative control deer, and infected versus starved deer), thus those estimates were omitted from Figure 4.

### 3.4 Behavioral changes in CWD‐infected deer occurred several months before death

**FIGURE 5:**
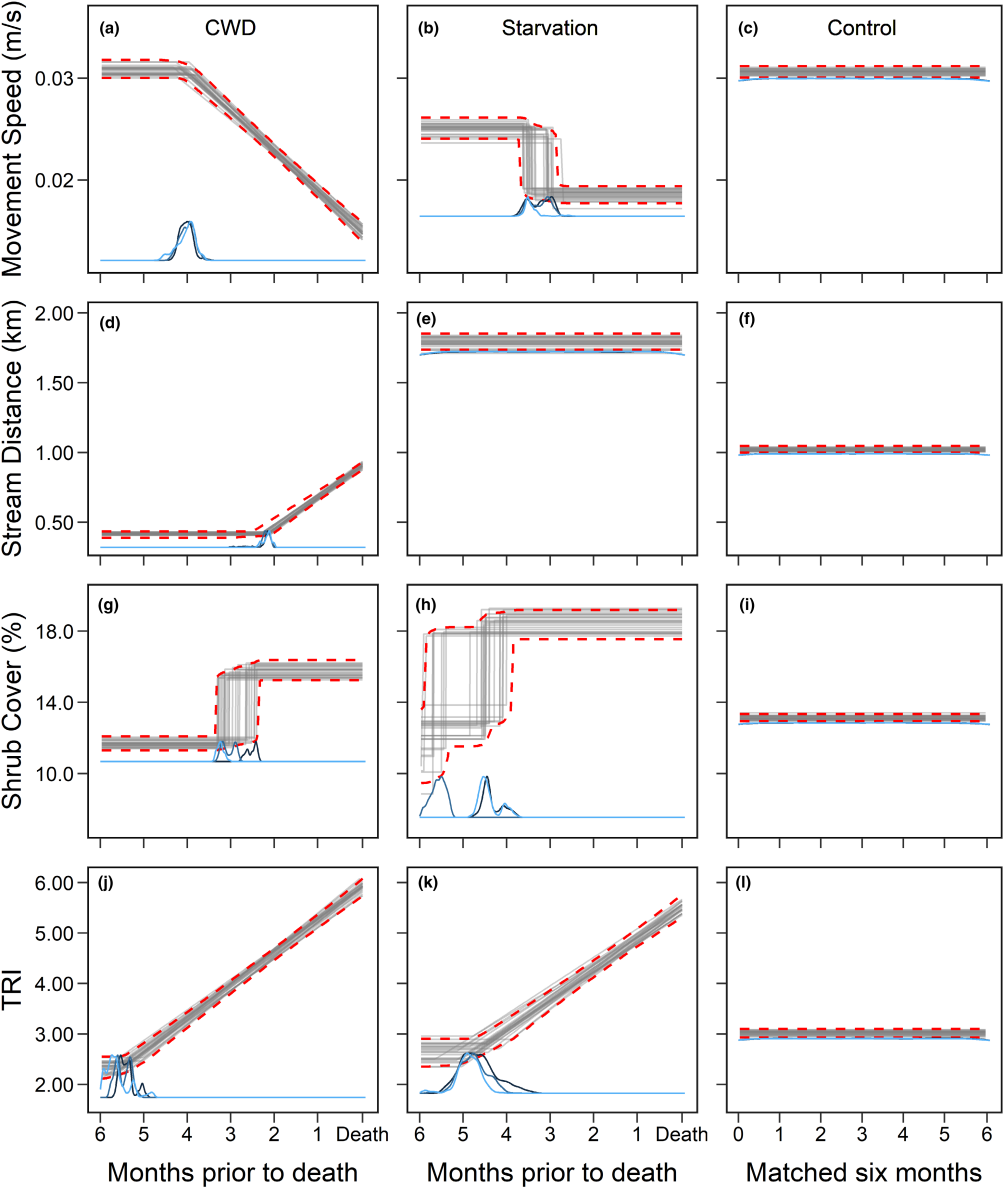
CWD‐infected mule deer (*n* = 18) often exhibited shifts in behavior during the final 6 months of life (panels a, d, g, j in the figure) within a sample of adult females collared during 2018–2022 in a CWD‐endemic region of central Wyoming, USA. Grey lines represent draws from the posterior distribution of the mean response and were derived from piecewise regressions fit using a Bayesian hierarchical modeling framework. We drew 2000 samples from each random effect (i.e., each individual deer). Red dashed lines depict the 95% credible intervals for the mean response. We conducted piecewise regression to identify the timing of behavioral shifts (i.e., change points) in the (a) movement and (d, g, j) habitat use of CWD‐infected deer. The posterior distribution for each change point is shown in blue on the x‐axis. To act as a positive control, we conducted piecewise regressions on the final 6 months of life for deer that succumbed to starvation (panels b, e, h, k in the figure; *n* = 8), as we posited that starved deer may behave similarly to CWD‐infected deer toward the end of life. Further, for each CWD‐infected and starved deer, we temporally matched data from a negative control deer (i.e., deer that died from causes other than CWD or starvation and those that survived the duration of the study) and conducted piecewise regression on this dataset. As depicted in (c, f, i, l), no change points were detected in the data from negative control deer (*n* = 26). TRI denotes terrain ruggedness index, with higher values signifying greater topographic heterogeneity.

CWD‐infected mule deer exhibited shifts in four different movement and habitat variables during the final 6 months of life (Figure 5). All remaining variables either displayed no behavioral change or piecewise regression models failed to converge. For movement speed, piecewise regression favored the model wherein CWD‐infected deer exhibited consistent speed (i.e., plateau) followed by a gradual decrease in speed as death from CWD approached (Figure 5a). Infected deer began to slow down on average 122 days prior to death (95% CI = 110–131) with variation across individuals (Appendix S1: Figure S1). In starved deer, by contrast, piecewise regression favored the model with an abrupt shift in movement speed (Figure 5b), which occurred 99 days prior to death (95% CI = 85–110). Notably, starved deer slowed to a lesser extent than CWD‐infected deer during the final 6 months of life. The null model with no behavioral change in movement speed was favored in negative control deer (Figure 5c). Overall, starved deer were already moving slower than CWD‐infected and negative control deer prior to 6 months before death.

CWD‐infected deer began to move away from streams approximately 65 days prior to death (95% CI = 58–73) (Figure 5d), whereas starved and negative control deer exhibited no behavioral change in proximity to stream (Figure 5e,f). Further, CWD‐infected deer were generally closer to streams than negative control deer and much closer than starved deer. For shrub cover, piecewise regression favored the model wherein CWD‐infected and starved deer abruptly shifted to use habitats with slightly higher shrub cover (Figure 5g,h). This shift occurred on average 88 days prior to death (95% CI = 72–100) in CWD‐infected deer and 143 days prior to death (95% CI = 116–175) in starved deer. Infected and starved deer also shifted to use more rugged topography (Figure 5j,k), a change that occurred around 165 days prior to death (95% CI = 152–179) in CWD‐infected deer and 143 days prior to death in starved deer (95% CI = 119–162). Null models with no behavioral change were favored in negative control deer for use of shrub cover and topographic ruggedness (Figure 5i,l). ELPD:SE ratios of second‐ranked models exceeded 4.5 for all model sets, indicating that each top model (i.e., those plotted in Figure 5) had superior accuracy.

## 4 DISCUSSION

CWD was the leading cause of mortality in adult female deer during our study. In the months preceding death from CWD, infected deer moved and used habitat differently than other deer, including deer that died from starvation. During later stages of disease, moreover, infected deer exhibited shifts in movement and habitat use. Overall, we demonstrate that infection alters host movement and habitat use in the cervid–CWD disease system, including in ways that can be distinguished from other pathologic states. Importantly, however, inference in this study is based on a relatively small number of animals from a limited spatial extent, such that our results may not generalize well to other regions/herds outside of our study area. Despite being limited in scope, our findings nevertheless provide valuable information to biologists and wildlife managers working to combat CWD. Thus, below we discuss how our results may relate to disease management, with emphasis on predator cleansing, environmental clean‐up, and potential hotspots for CWD transmission. We also further discuss the limitations of our study and outline intriguing lines of future inquiry.

### 4.1 CWD‐induced shifts in habitat use may help target environmental cleanup efforts

During later stages of infection, CWD‐positive deer used more rugged terrain in lower elevation areas with slightly higher tree and shrub cover than uninfected deer, though differences in the use of tree and shrub cover were relatively small. Although CWD contamination of the environment via prion shedding in cervid excreta can occur as early as 6 months post‐infection (Plummer et al., 2017), animals are likely most infectious (i.e., shedding prions most rapidly) during later stages of clinical disease. Thus, lower‐lying areas with higher cover could be hotspots for direct and/or indirect transmission of CWD in our study areas, assuming susceptible animals also use such habitats. In agreement with our findings, prior work suggested that landscape features such as forest patches and lower elevation areas closer to rivers were associated with higher risk of CWD (Evans et al., 2016; O'Hara Ruiz et al., 2013). For instance, at our study sites, such areas in the Upper Powder River include habitat directly adjacent to the Middle and North Fork of the Powder River, Steel Creek, Tex Springs Creek, and the Middle and North Fork of Crazy Woman Creek, and in Bates Hole include habitat directly adjacent to Stinking Creek, Clear Fork Muddy Creek, Smith Creek, Skeen Creek, Corral Creek, and Bates Creek. All areas described above represent areas where CWD‐infected deer spent considerable time throughout the winters of our study period.

Managers could modify or clean‐up such areas to reduce their role as environmental reservoirs of CWD (Escobar et al., 2020). However, the effectiveness and scalability of environmental clean‐up for CWD prions remains unclear, and current options would be time‐ and cost‐intensive (e.g., prescribed fire, habitat restoration; Haley & Hoover, 2015). Several novel techniques have been proposed to remove prions from contaminated environments, including microorganismal composting and protease treatment (Dickinson et al., 2009; Smith et al., 2011; Xu et al., 2013), though more work is needed to assess the risks and practical means for decontamination. Our results on host habitat use during clinical stages of CWD infection could provide valuable insight into where decontamination efforts may be best piloted. Additionally, determining the extent to which disease‐induced shifts in space use result in infected deer congregating in shared habitats (i.e., overlapping in space and time) could further refine where CWD hotspots are most likely to occur.

Contrary to our predictions, CWD‐positive deer moved away from streams around 2 months prior to death, after being relatively close to riparian habitat 2–6 months before death. Infected deer presumably seek out riparian habitat in search of drinking water due to extreme thirst caused by clinical disease (Williams & Young, 1993). Polydipsia may associated with a discrete stage of infection, however, such that excessive thirst is halted by 2 months before death and/or overridden by other pathophysiology (Miller et al., 1998). Further, some drainages in our study area are ephemeral. Given ephemerality in our system, coupled with a relatively low sample size of CWD‐infected deer, our finding that infected deer move away from riparian areas around 2 months before death may not generalize to regions/herds outside of our study area and/or may constitute a spurious result. Nevertheless, our results overall suggest that CWD‐positive deer used areas closer to streams than starved and negative control deer. Thus, CWD‐infected deer may be shedding prions near streams during clinical stages of disease, which underscores riparian zones as potential hotspots for environmental contamination and thus indirect transmission of CWD in our study area.

### 4.2 CWD‐induced shifts in movement speed may support predator‐mediated disease management

Infected deer began to slow down around 4 months prior to death from CWD and moved slower than many of their uninfected conspecifics during the final 2 months of life. Thus, if predators can detect and exploit slower prey, then predators may selectively remove infectious individuals, possibly improving the health of the host population (Packer et al., 2003). Indeed, predators would have the greatest impact on decreasing the prevalence of CWD if the most infectious individuals in a prey population are also the most vulnerable to predation (Brandell et al., 2022). We could not directly test whether CWD‐positive animals were more susceptible to predation, though future work could assess the proportion of predation deaths that exhibited movement behavior indicative of CWD infection.

Most kills in our study area were by cougars (20 of 27 kills). Indeed, multiple studies provide evidence that cougars selectively prey upon CWD‐infected mule deer (e.g., Krumm et al., 2010; Miller et al., 2008). In a model developed using empirical data from the Greater Yellowstone Ecosystem, cougar decreased CWD outbreak size substantially; however, this effect was driven by their ability to select prey in the late stages of CWD disease progression (Brandell et al., 2022). By demonstrating that infected deer slow down several months prior to death and move slower and use different habitat types than many uninfected conspecifics, our results provide evidence for differences in prey behavior on which predators could potentially base their selectivity. Yet, high infection rates can remain despite selective predation of CWD‐infected mule deer by cougars, possibly due to the increased congregation of prey to avoid predators (Miller et al., 2008). Thus, while our results help establish a behavioral mechanism to explain how predators may detect and select infected prey, future studies could build from our work to determine the strength of selectively needed to reduce CWD prevalence and transmission in natural systems (Brandell et al., 2022).

### 4.3 Capitalizing on CWD‐induced behavioral shifts to assess compensatory versus additive predation

For disease systems wherein infection alters host movement and habitat use in observable ways—as we show for the cervid–CWD system in this study—predicting infection status from telemetry data represents a potentially powerful yet relatively unexplored diagnostic tool for disease management (Dougherty et al., 2018). For instance, identifying the precise timing of infection constitutes a fundamental limitation in wildlife disease studies. Disease data are collected discretely and usually require handling animals, making temporal conclusions on the timing of pathogen exposure and stage of disease progression difficult to ascertain. By contrast, telemetry data from GPS collars are collected continuously. A predictive model that can reliably infer disease status from GPS data could therefore leverage continuous movement data to reveal information on the timing of clinical infection. Such a predictive model also could be useful to supplement field and laboratory diagnostics or when mortalities cannot be quickly retrieved to assess cause‐specific mortality. Specifically, identifying individuals that are sick before predation events could help to assess the extent to which disease mortality is compensatory versus additive with predation.

### 4.4 Caveats

Disease‐induced behavioral shifts may occur prior to 6 months before death in some hosts infected with CWD (Williams, 2005). We believe this could have been the case for several infected deer that moved at relatively slow speeds during the last 6 months of life, without exhibiting behavioral shifts during this period (Appendix S1: Figure S1). Limiting our inference to 6 months prior to death therefore likely constituted a limitation of our study, albeit necessary to maintain adequate sample sizes for analysis. Still, our inference was based on a relatively small number of animals inhabiting a narrow spatial extent, such that our results may not generalize well to other regions/herds outside of our study area.

Our analysis included only adult females, which limited our inference given that male and female mule deer can move and select habitat differently (Rodgers et al., 2021). For instance, males often have larger home ranges than females (Relyea et al., 2000), potentially increasing risk of exposure to CWD via greater encounter rates with infected individuals and/or contaminated environments. Indeed, male mule deer are often 1.5–3 times more likely to be infected with CWD compared to females (DeVivo et al., 2017; Miller et al., 2008). Although the source of this variation remains unknown, it is most likely that behavioral, not physiological, differences between males and females drive greater prevalence in males (Rogers et al., 2022). Still, it remains unclear whether male‐biased prevalence results from higher male‐to‐male transmission (e.g., high contact rates within bachelor groups outside of mating season) or higher female‐to‐male transmission (e.g., reproductive behaviors during the mating season). Notably, simulations suggest that male‐biased harvests were ineffective under high female‐to‐male transmission (Rogers et al., 2022). Female‐biased harvests were able to limit CWD transmission in this scenario, though managers must balance the epidemiological benefit of female harvests with demographic costs as the viability of many cervid populations is most sensitive to adult female survival (Raithel et al., 2007). Clearly, CWD transmission between sexes is critical for determining the intensity of sex‐specific harvests and will be an important line of inquiry for future work aimed at informing disease management (Potapov et al., 2016).

Although we temporally matched behavioral comparisons between groups (CWD, starvation, negative control) to account for differences in behavior throughout the year (e.g., quicker movements during migration) and to correct for potential biases if animals died more often during specific times of year, seasonal differences in space use behavior still could have influenced our results to some degree. For instance, perhaps adult females move less during the fawning season, such that behavioral differences between CWD‐infected deer and healthy deer were less pronounced during this period. Data used in final analyses had substantial coverage during each month of the year (i.e., similar number of observations in each month), however, providing us confidence that seasonal differences in behavior did not bias our results due to temporal gaps in the data (Appendix S1: Figure S2). Still, starved deer had relatively more data during winter months, which could have influenced our results given deer may move more/less (or select specific habitats) during this period (Appendix S1: Figure S2c).

We found that CWD‐infected deer migrated at similar proportions to all other deer at both Bates Hole and the Upper Powder River. In this analysis, however, we included all data from CWD‐infected animals, not just the final 6 months of life (to ensure we captured the migration period). Thus, migration rates could be biased given that CWD often includes prolonged latent periods of infection (Miller et al., 2000), such that conspicuous changes in movement behavior may not manifest until later stages of clinical disease (e.g., a few months prior to death). Further, social dynamics could have confounded our results to some extent, if CWD‐infected deer were clustered within social groups that selected for particular habitat types. CWD‐infected deer used spatially isolated winter ranges (see above), however, providing us confidence that most animals did not belong to the same social group. Still, sociality may have played some role in our results. Although quantifying group dynamics was outside of the scope of this work, future work may benefit from integrating spatial and social networks into wildlife disease research (Albery et al., 2021).

Finally, uncertainty in cause of death could have influenced our findings. Cause of death was determined for each mortality based on combined interpretation of field observations, laboratory test results, and postmortem evaluation by a board‐certified pathologist, including gross assessment and histopathology. Our CWD mortality category included deer that tested positive for CWD and did not have detectable evidence of a concurrent pathologic process, as well as deer that tested positive for CWD and were killed by a predator, based on the assumption that CWD predisposes to predation. When possible, we used histopathology as corroborating evidence of clinical CWD. However, many cases were either scavenged or autolyzed, and we could not confirm lesions in all cases. Further, our assessment of predation was based on a combination of field observations (e.g., presence of tracks or scat, carcasses cached or not cached) and when possible, gross assessment of carcasses for the presence of puncture wounds. Species of predator was inferred based on wound pattern and intercanine width (Verzuh et al., 2018); however, not all carcasses were recovered for transport to the laboratory and assessment by a pathologist, and some were recovered in poor postmortem condition. While efforts were made to use all available information to determine cause of death, not all information was obtainable for every mortality.

## 5 CONCLUSIONS

Mule deer populations have experienced widespread declines across western North America (Bergman et al., 2015; Bishop et al., 2009), motivating a need to better understand cause‐specific mortality. Herein, we describe how infection with CWD alters the movement and habitat use of mule deer in the months preceding death. Such infection‐induced behavioral shifts may allow for the development of predictive models of disease status from movement data, which will be useful to supplement field and laboratory diagnostics when assessing cause‐specific mortality. CWD and predation often represent major sources of mortality in mule deer populations (DeVivo et al., 2017; Miller et al., 2008). Indeed, CWD and predation were the leading causes of death in our study. Yet, there is little research to disentangle the extent to which disease mortalities are compensatory versus additive with predation (e.g., many predation deaths may be facilitated by disease as a predisposing factor). A predictive model of disease status based on movement data would allow further parsing of predation deaths into individuals that were healthy versus sick prior to being consumed.

More generally, emerging infectious diseases threaten the conservation of global biodiversity (Jones et al., 2008). Understanding how organisms respond to disease will be critical to guide proactive management. Our overall approach for detecting the timing of infection‐induced shifts in movement behavior may be useful in application to other disease systems to better understand how animals respond to infection with emerging pathogens.

## AUTHOR CONTRIBUTIONS

**Gabriel M. Barrile:** Conceptualization (lead); data curation (equal); formal analysis (lead); funding acquisition (supporting); investigation (lead); methodology (equal); project administration (lead); resources (lead); validation (lead); visualization (lead); writing – original draft (lead); writing – review and editing (lead). **Paul C. Cross:** Conceptualization (supporting); funding acquisition (equal); investigation (supporting); project administration (equal); resources (supporting); writing – review and editing (supporting). **Cheyenne Stewart:** Conceptualization (supporting); data curation (supporting); funding acquisition (equal); investigation (supporting); project administration (equal); resources (supporting); writing – review and editing (supporting). **Jennifer Malmberg:** Conceptualization (supporting); data curation (supporting); funding acquisition (equal); investigation (supporting); project administration (equal); resources (supporting); writing – review and editing (supporting). **Rhiannon P. Jakopak:** Conceptualization (supporting); data curation (supporting); funding acquisition (supporting); investigation (equal); project administration (equal); resources (supporting); writing – review and editing (supporting). **Justin Binfet:** Conceptualization (supporting); data curation (supporting); funding acquisition (equal); investigation (supporting); project administration (equal); resources (supporting); writing – review and editing (supporting). **Kevin L. Monteith:** Conceptualization (supporting); data curation (supporting); funding acquisition (equal); investigation (supporting); project administration (equal); resources (supporting); writing – review and editing (supporting). **Brandon Werner:** Conceptualization (supporting); data curation (supporting); funding acquisition (equal); investigation (supporting); project administration (equal); resources (supporting); writing – review and editing (supporting). **Jessica Jennings‐Gaines:** Conceptualization (supporting); data curation (supporting); funding acquisition (equal); investigation (supporting); project administration (equal); resources (supporting); writing – review and editing (supporting). **Jerod A. Merkle:** Conceptualization (supporting); data curation (supporting); funding acquisition (equal); investigation (supporting); project administration (equal); resources (supporting); writing – review and editing (supporting).

## CONFLICT OF INTEREST STATEMENT

The authors declare no conflict of interest.

## Supporting information


Appendix S1.

## Data Availability

Data and code used in analyses are archived in the Dryad Digital Repository as “Private for Peer Review” and are accessible to reviewers via this link: https://datadryad.org/stash/share/8iMKsYmuKtc0eznYepIBLISxi‐E658OkDNmuTjYHIcA.
