# Supplementary material for: Chronic wasting disease alters the movement behavior and habitat use of mule deer during clinical stages of infection
Source: Ecol Evol. 2024 May 21;14(5):e11418. doi: 10.1002/ece3.11418 (PMC11108800; doi:10.1002/ece3.11418)
Supplement: Supplementary file 1 — Appendix S1. [file ECE3-14-e11418-s001.docx]

**Appendix S1**

**Journal Name:** *Ecology and Evolution*

**Manuscript Title**: Chronic wasting disease alters the movement behavior and habitat use of mule deer during clinical stages of infection

**List of Authors:**

Gabriel M. Barrile, Paul C. Cross, Cheyenne Stewart, Jennifer Malmberg, Rhiannon P. Jakopak, Justin Binfet, Kevin L. Monteith, Brandon Werner, Jessica Jennings-Gaines, and Jerod A. Merkle

**Corresponding Author Email:** [gbarrile15@gmail.com](mailto:gbarrile15@gmail.com)**Table S1.** Pearson’s correlation coefficients of movement and habitat metrics derived from GPS locations of adult female mule deer (*n* = 179) collared during 2018–2022 in a CWD-endemic region of central Wyoming, USA. Bolded numbers denote bivariate correlations greater than |0.60|, which we considered to be highly correlated and thus were included in comparisons of univariate models to determine which correlated variable(s) to retain in final analysis.

|  | *speed* | *max* | *disp* | *ta* | *biom* | *shrub* | *tree* | *herb* | *elev* | *slope* | *tri* | *road* | *stream* | *water* |
| --- | --- | --- | --- | --- | --- | --- | --- | --- | --- | --- | --- | --- | --- | --- |
| *speed* | 1.00 |  |  |  |  |  |  |  |  |  |  |  |  |  |
| *max* | **0.79** | 1.00 |  |  |  |  |  |  |  |  |  |  |  |  |
| *disp* | **0.85** | **0.73** | 1.00 |  |  |  |  |  |  |  |  |  |  |  |
| *ta* | 0.00 | 0.00 | 0.01 | 1.00 |  |  |  |  |  |  |  |  |  |  |
| *biom* | 0.09 | -0.02 | 0.03 | 0.00 | 1.00 |  |  |  |  |  |  |  |  |  |
| *shrub* | -0.08 | 0.00 | -0.02 | 0.00 | -0.35 | 1.00 |  |  |  |  |  |  |  |  |
| *tree* | -0.03 | -0.03 | -0.02 | 0.01 | -0.26 | -0.18 | 1.00 |  |  |  |  |  |  |  |
| *herb* | 0.10 | 0.01 | 0.04 | 0.00 | **0.83** | -0.29 | -0.53 | 1.00 |  |  |  |  |  |  |
| *elev* | -0.01 | 0.09 | 0.01 | 0.01 | -0.39 | 0.17 | 0.49 | -0.41 | 1.00 |  |  |  |  |  |
| *slope* | -0.16 | -0.05 | -0.09 | 0.01 | -0.37 | 0.19 | 0.13 | -0.36 | 0.28 | 1.00 |  |  |  |  |
| *tri* | -0.16 | -0.05 | -0.09 | 0.00 | -0.38 | 0.19 | 0.11 | -0.36 | 0.25 | **0.94** | 1.00 |  |  |  |
| *road* | -0.06 | -0.04 | -0.02 | 0.00 | -0.11 | 0.07 | -0.01 | -0.10 | -0.05 | 0.16 | 0.16 | 1.00 |  |  |
| *stream* | -0.03 | -0.01 | -0.01 | 0.00 | -0.23 | 0.18 | -0.09 | -0.13 | 0.06 | 0.13 | 0.13 | 0.16 | 1.00 |  |
| *water* | -0.01 | 0.01 | -0.01 | 0.01 | -0.01 | 0.05 | 0.03 | -0.02 | 0.15 | 0.31 | 0.09 | 0.40 | **0.64** | 1.00 |

*Notes:* *speed* = mean speed; *max* = maximum speed; *disp* = displacement; *ta* = turning angle; *biom* = herbaceous biomass of forbs and grasses; *shrub* = percent shrub cover; *tree* = percent tree cover; *herb* = percent cover of forbs and grasses; *elev* = elevation; *slope* = slope; *tri* = terrain ruggedness index; *road* = distance to nearest road; *stream* = distance to nearest linear water feature (e.g., streams, canals); *water* = distance to nearest water body (e.g., lakes, reservoirs). The derivation of each variable is described in detail within the main text of the manuscript.

**Table S2.** Akaike information criterion (AIC) scores from univariate conditional logistic regression models comparing the behavior of CWD-infected deer (*n* = 18) to negative control deer (*n* = 125). We compared univariate models to determine which variable in each correlated set to retain in final analyses. Subscript numbers next to AIC in column names denote the month before death from CWD to which each model was fit. For instance, AIC_12_ indicates that the model was fit using data 1–2 months before death from CWD. Data from negative control animals were temporally matched to data from CWD-infected deer prior to model fitting. Empty rows in the table separate each set of correlated variables. Bolded numbers denote the lowest AIC score.

| Correlated Variables | AIC_01_ | AIC_12_ | AIC_23_ | AIC_34_ | AIC_45_ | AIC_56_ |
| --- | --- | --- | --- | --- | --- | --- |
| *speed* | **4021.81** | **3985.24** | **4053.48** | **4050.20** | **4055.37** | **4107.20** |
| *disp* | 4107.14 | 4019.29 | 4098.23 | 4078.74 | 4067.72 | 4124.89 |
| *max* | 4140.37 | 4028.37 | 4078.78 | 4080.57 | 4057.37 | 4133.45 |
|  |  |  |  |  |  |  |
| *biom* | **4214.81** | **4097.23** | 4106.37 | **4096.29** | **4065.24** | **4088.68** |
| *herb* | 4219.56 | 4099.35 | **4105.52** | 4096.33 | 4069.60 | 4113.00 |
|  |  |  |  |  |  |  |
| *tri* | **4190.94** | **4087.53** | 4097.81 | **4084.59** | 4046.85 | **4071.51** |
| *slope* | 4207.42 | 4093.29 | **4096.69** | 4086.70 | **4046.34** | 4074.52 |
|  |  |  |  |  |  |  |
| *stream* | **4187.18** | **4061.64** | **4006.51** | **4002.59** | **4022.19** | **4030.67** |
| *water* | 4212.61 | 4105.02 | 4090.24 | 4096.54 | 4086.53 | 4139.76 |

*Notes:* *speed* = mean speed; *disp* = displacement; *max* = maximum speed; *biom* = herbaceous biomass of forbs and grasses; *herb* = percent cover of forbs and grasses; *tri* = terrain ruggedness index; *slope* = slope; *stream* = distance to nearest linear water feature (e.g., streams, canals); *water* = distance to nearest water body (e.g., lakes, reservoirs). The derivation of each variable is described in detail within the main text of the manuscript. In final models, we retained *speed*, *biom*, *tri*, and *stream* from the sets of correlated variables displayed in the table. These variables were retained because they produced the lowest AIC scores in most if not all six models (one for each month before death) comparing the behavior of CWD-infected deer to negative control deer (i.e., animals that died from causes other than CWD and starvation and deer that survived the duration of the study).


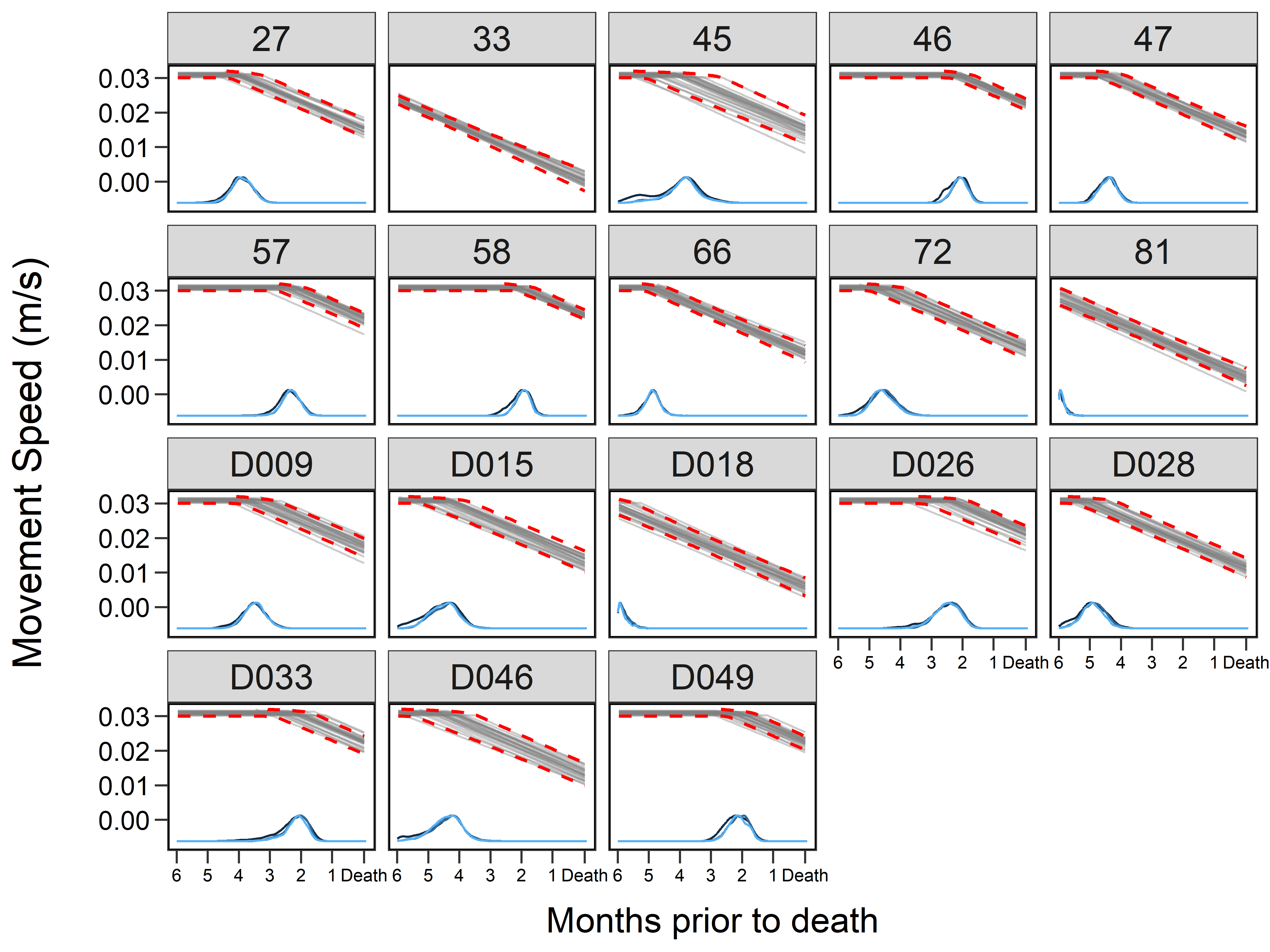


**Figure S1.** Individual-specific change points for each CWD-positive mule deer (*n* = 18) in relation to mean daily movement speed during the final six months of life within a sample of adult females collared during 2018–2022 in central Wyoming, USA. Grey lines represent draws from the posterior distribution of the mean response and were derived from piecewise regressions fit using a Bayesian hierarchical modeling framework. Red dashed lines depict the 95% credible intervals for the mean response. The posterior distribution for each change point is shown in blue on the x-axis. Notably, several posterior distributions for individual change points fell earlier than six months before death (e.g., deer 33, deer 81, deer D018), suggesting that those individuals likely began to slow down prior to six months before death.


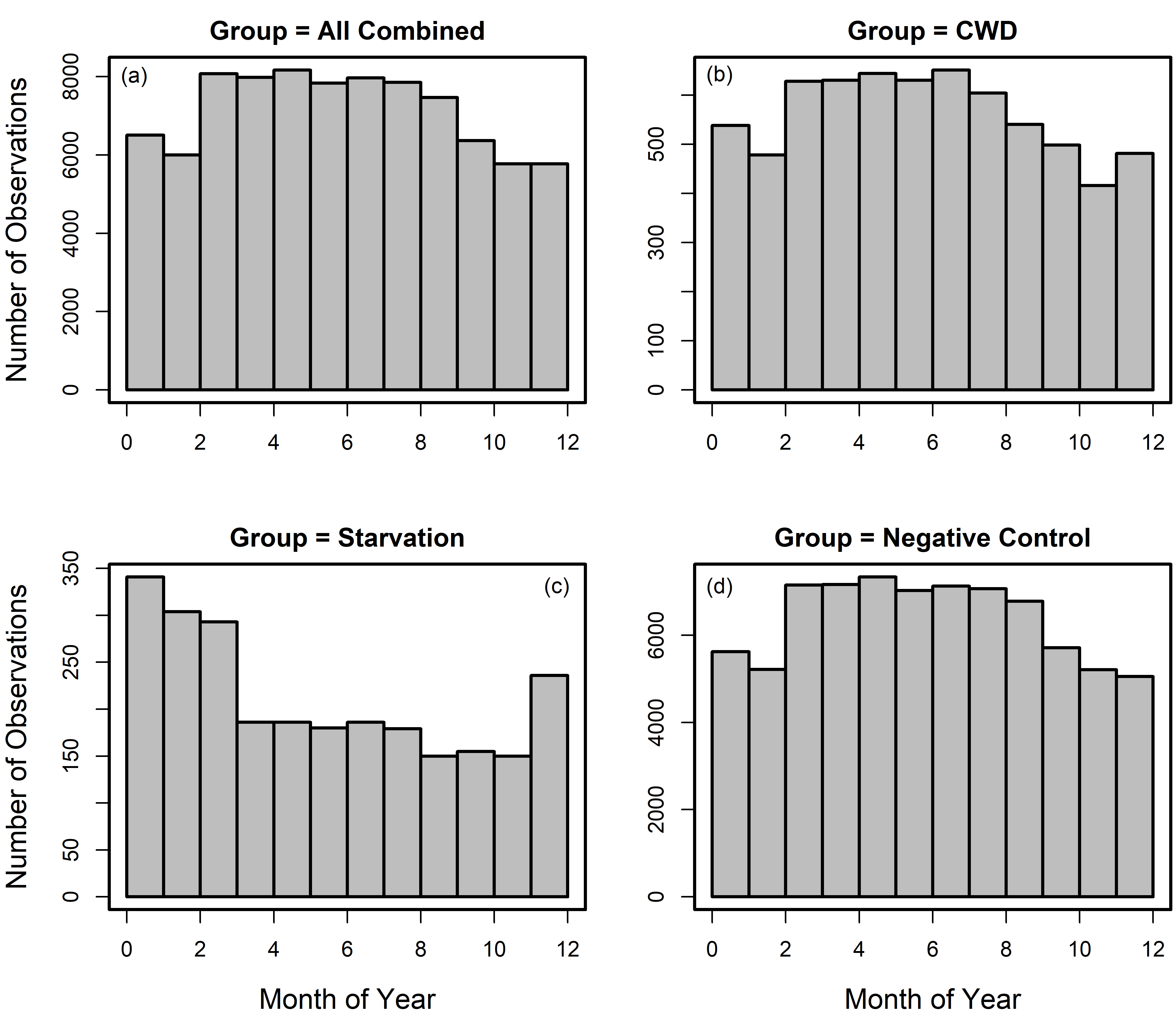


**Figure S2.** Histograms displaying the number of observations (i.e., GPS locations) used in final analyses comparing the space use of adult female mule deer that (b) died from CWD, (c) died from starvation, and (d) negative control animals, which included deer that died from causes other than CWD and starvation and deer that survived the duration of the study. Panel (a) displays the raw data for all groups (b–d) combined. Adult female mule deer were collared during 2018–2021 in the Upper Powder River and during 2021–2022 at Bates Hole in a CWD-endemic region of central Wyoming, USA.
